# Explicit Theoretical Analysis of How the Rate of Exocytosis Depends on Local Control by Ca^2+^ Channels

**DOI:** 10.1155/2018/5721097

**Published:** 2018-11-14

**Authors:** Francesco Montefusco, Morten Gram Pedersen

**Affiliations:** ^1^Department of Information Engineering, University of Padova, Padova, Italy; ^2^Department of Mathematics “Tullio Levi-Civita”, University of Padova, Padova, Italy; ^3^Padova Neuroscience Center, University of Padova, Padova, Italy

## Abstract

Hormones and neurotransmitters are released from cells by calcium-regulated exocytosis, and local coupling between Ca^2+^ channels (CaVs) and secretory granules is a key factor determining the exocytosis rate. Here, we devise a methodology based on Markov chain models that allows us to obtain analytic results for the expected rate. First, we analyze the property of the secretory complex obtained by coupling a single granule with one CaV. Then, we extend our results to a more general case where the granule is coupled with *n* CaVs. We investigate how the exocytosis rate is affected by varying the location of granules and CaVs. Moreover, we assume that the single granule can form complexes with inactivating or non-inactivating CaVs. We find that increasing the number of CaVs coupled with the granule determines a much higher rise of the exocytosis rate that, in case of inactivating CaVs, is more pronounced when the granule is close to CaVs, while, surprisingly, in case of non-inactivating CaVs, the highest relative increase in rate is obtained when the granule is far from the CaVs. Finally, we exploit the devised model to investigate the relation between exocytosis and calcium influx. We find that the quantities are typically linearly related, as observed experimentally. For the case of inactivating CaVs, our simulations show a change of the linear relation due to near-complete inactivation of CaVs.

## 1. Introduction

Molecules, e.g., neurotransmitters and proteins, are released from the cell by exocytosis [1]. In this paper, we focus on regulated exocytosis in the endocrine cells that release different kinds of hormones regulating various physiological processes [2]. When hormone secretion is defectively regulated, several diseases may develop. For example, in diabetes, the two main pancreatic hormones, insulin and glucagon, are not released appropriately for fine-tuning glucose homeostasis [3, 4]. Therefore, it is crucial to achieve a better understanding of the main mechanisms underlying hormone exocytosis that determines the control of different physiological processes.

In most endocrine cells, the hormones are contained in secretory granules that, in response to a series of cellular mechanisms culminating with an increase in the intracellular Ca^2+^ levels, fuse with the cell membrane and release the hormone molecules. The main mechanisms regulating hormone exocytosis are shared with exocytosis of synaptic vesicles underlying neurotransmitter release in neurons [1, 5]. The granules contain v-SNARE proteins that can form the so-called SNARE complexes with t-SNAREs inserted in the cell membrane [1]. SNARE complexes interact with other proteins, notably, Ca^2+^-sensing proteins such as synaptotagmins, which trigger exocytosis upon Ca^2+^ binding. Therefore, the local Ca^2+^ concentration at the Ca^2+^ sensor of the exocytotic machinery is a key factor determining the probability rate of exocytosis of the secretory granule [6].

Recently, we have devised a detailed model of Ca^2+^ dynamics and exocytosis for the glucagon-secreting pancreatic alpha-cells and showed how exocytosis is dependent on calcium dynamics, in particular, on calcium levels surrounding the Ca^2+^ channels (CaVs) [7], the so-called nanodomains [8]. Here, in order to characterize the local interactions between the single granule and the surrounding CaVs, we will exploit a strategy that is similar to the methodology devised in our recent paper to describe the large conductance BK potassium current that is controlled locally by CaVs [9]. We showed that the number and the type of CaVs coupled with the BK channel affect the electrical activity of neurons and other excitable cells, such as pancreatic beta-cells and pituitary cells. Therefore, we will implement mathematical modelling for characterizing the local interactions between granules and CaVs and, specifically, Markov chain models that could provide important insight into the exocytosis rate. In particular, by using the Markov chain theory [10], we will achieve analytic results for the expected rate and show how coupling different numbers and types of CaVs with the granule determines different responses.

## 2. Methods

### 2.1. CaV Channel Model

**Figure 1:**
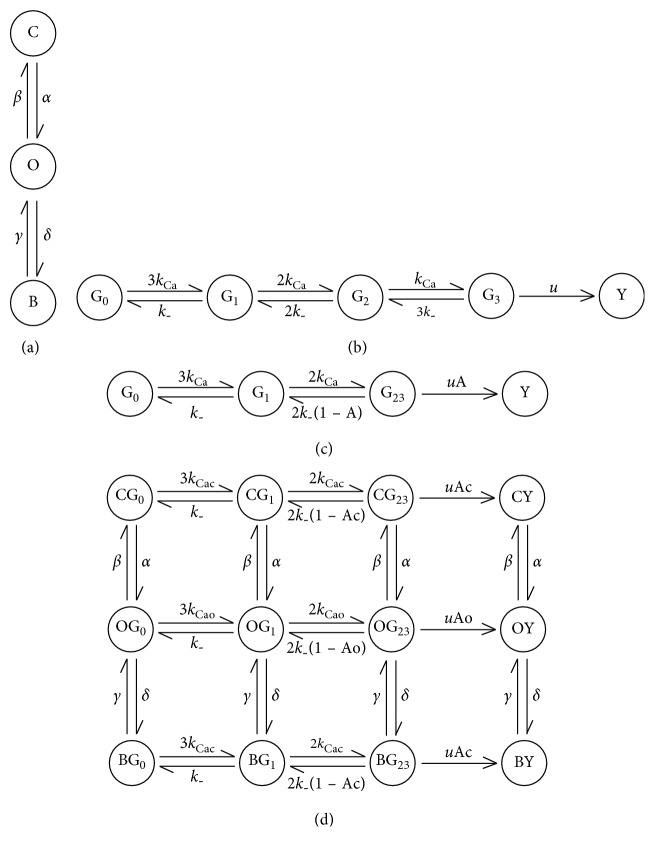
Markov chain models for Ca^2+^ channel (CaV), exocytosis of single granule and granule-CaV complex. (a) Markov chain model for CaV, where C is the closed state, O the open state, and B the inactivated or blocked state. (b) Markov chain model for exocytosis of a single granule adjacent to the plasma membrane, where *G*_0_ correspond to the state with no bound Ca^2+^ ions, *G*_1_ with one, *G*_2_ with two, and *G*_3_ with three. (c) Markov chain model for the approximated exocytosis model where the dynamics of states *G*_2_ and *G*_3_ are described by the auxiliary variable *G*_23_ using quasi-steady state approximation for the corresponding ODE model. (d) Markov chain model for the granule-CaV complex where the granule dynamics are described by the model shown in panel (c) and the CaV dynamics by the model shown in panel (a).

We model the Ca^2+^ channel by using the 3-state Markov chain of Figure 1(a), where *C* corresponds to the closed state, *O* to the open state, and *B* to the inactivated (blocked) state of the calcium channel [11]. Then, the CaV model takes values in the state space *S*={*C*, *O*, *B*} and its transition rate or generator matrix *M*_CaV_ is given by

(1) $$\begin{array}{c} M_{\text{CaV}}=\left[\begin{array}{ccc} -\alpha & \alpha & 0\\ \beta & -\beta -\delta & \delta \\ 0 & \gamma & -\gamma \end{array}\right], \end{array}$$

where *α* and *β* represent the voltage-dependent Ca^2+^ channel opening rate and closing rate, respectively, and have the following forms:

(2) $$\begin{array}{c} \alpha \left(V\right)=\alpha_{0}e^{-\alpha_{1}V},\\ \beta \left(V\right)=\beta_{0}e^{-\beta_{1}V}. \end{array}$$

The rate for channel inactivation, *δ*, is Ca^2+^-dependent and has the following form:

(3) $$\begin{array}{c} \delta =\delta_{0}\times \left[Ca_{\text{CaV}}\right], \end{array}$$

where *Ca*_CaV_ is the Ca^2+^ concentration at the Ca^2+^ sensor for inactivation and is given using reaction-diffusion theory [8, 12, 13] by

(4) $$\begin{array}{c} {\text{Ca}}_{\text{CaV}}=\frac{i_{{\text{Ca}}_{\max}}}{8\pi r_{\text{Ca}}D_{\text{Ca}}F}\exp\left[\frac{-r_{\text{Ca}}}{\sqrt{D_{\text{Ca}}/\left(k_{B}^{+}\left[B_{\text{total}}\right]\right)}}\right], \end{array}$$

where $i_{{\text{Ca}}_{\max}}={\bar{g}}_{\text{Ca}}\left(V-V_{\text{Ca}}\right)$ is the single-channel Ca^2+^ current with ${\bar{g}}_{\text{Ca}}$ the single-channel conductance and *V*_Ca_ the reverse potential, and *r*_Ca_ represents the distance of the sensor for Ca^2+^-dependent inactivation from the channel pore. Finally, *γ* is the constant reverse reactivation rate.  Table 1 reports the parameter values for the CaV model defined by above equations.

The deterministic description of the 3-state Markov chain model for the CaV channel is given by the following ODE system:

(5) $$\begin{array}{c} \frac{dc}{dt}=\beta o-\alpha c,\\ \frac{do}{dt}=\alpha c+\gamma b-\left(\beta +\delta \right)o,\\ b=1-c-o=1-h, \end{array}$$

where the italic lowercase letters represent the corresponding state variables of the ODE model (*h* represents the fraction of Ca^2+^ channels not inactivated).

Finally, in order to investigate the relationship between exocytosis and Ca^2+^ loading, we compute the total charge entering via the Ca^2+^ channel at a given step voltage with time window, *t*_s_, as

(6) $$\begin{array}{c} Q_{\text{Ca}}=\int_{0}^{t_{\text{s}}}o\left(\tau \right)\cdot i_{{\text{Ca}}_{\max}}\;d\tau . \end{array}$$

### 2.2. Exocytosis Model

We assume a single granule, adjacent to the plasma membrane and primed for exocytosis, that can be in one of four different states depending on the number of Ca^2+^ ions bound to the Ca^2+^ sensor on the granule, likely synaptotagmin [14]: in *G*_0_ with no bound Ca^2+^ ions, or in *G*_1_ with one, or in *G*_2_ with two, or in *G*_3_ with three bound ions. Once it is in *G*_3_, the granule can fuse with the membrane and release its hormone content, assuming the final state *Y* [6, 15]. Therefore, we use a five-state Markov chain model for describing exocytosis as shown in Figure 1(b), where the model takes values in the state space *S*={*G*_0_, *G*_1_, *G*_2_, *G*_3_, *Y*}, and its transition rate or generator matrix *M*_G_ is given by

(7) $$\begin{array}{c} M_{\text{G}}=\left[\begin{array}{ccccc} -3k_{\text{Ca}} & 3k_{\text{Ca}} & 0 & 0 & 0\\ k_{-} & -2k_{\text{Ca}}-k_{-} & 2k_{\text{Ca}} & 0 & 0\\ 0 & 2k_{-} & -k_{\text{Ca}}-2k_{-} & k_{\text{Ca}} & 0\\ 0 & 0 & 3k_{-} & -u-3k_{-} & u\\ 0 & 0 & 0 & 0 & 0 \end{array}\right], \end{array}$$

where

(8) $$\begin{array}{c} k_{\text{Ca}}=k_{+}\times \left[{\text{Ca}}_{\text{G}}\right] \end{array}$$

represents the Ca^2+^ binding rate, with Ca_G_ the Ca^2+^ concentration at the granule sensor given by Equation (4) with *r*=*r*_G_ being the distance from the CaV to the Ca^2+^ sensor on the granule. In the following, the distance from the CaV to the granule means the distance from the CaV to the Ca^2+^ sensor on the granule, which will be of the order of tens of nm. For comparison, secretory granules have diameters on the order 100–500 nm [16–19]. We assume a constant number of Ca^2+^ sensor molecules, which is therefore included in the binding parameter *k*_Ca_. The parameter *k*_−_ is the unbinding rate, and *u* is the fusion rate.  Table 1 reports the parameter values.

The deterministic description of the 5-state Markov chain model for exocytosis is given by the following ODE system:

(9) $$\begin{array}{c} \frac{dg_{0}}{dt}=-3k_{\text{Ca}}g_{0}+k_{-}g_{1}, \end{array}$$

(10) $$\begin{array}{c} \frac{dg_{1}}{dt}=-\left(2k_{\text{Ca}}+k_{-}\right)g_{1}+3k_{\text{Ca}}g_{0}+2k_{-}g_{2}, \end{array}$$

(11) $$\begin{array}{c} \frac{dg_{2}}{dt}=-\left(k_{\text{Ca}}+2k_{-}\right)g_{2}+2k_{\text{Ca}}g_{1}+3k_{-}g_{3}, \end{array}$$

(12) $$\begin{array}{c} \frac{dg_{3}}{dt}=-\left(u+3k_{-}\right)g_{3}+k_{\text{Ca}}g_{2}, \end{array}$$

(13) $$\begin{array}{c} y=1-g_{0}-g_{1}-g_{2}-g_{3}. \end{array}$$

For the above ODE model of Equations (9)–(13), we exploit quasi steady-state approximation for state *g*_3_, since its dynamics are fastest (the value of *u* is much higher than those of the other parameters). Then, by renaming the state variables as

(14) $$\begin{array}{c} g_{23}=g_{2}+g_{3}, \end{array}$$

by setting Equation (12) equal to zero yielding

(15) $$\begin{array}{c} g_{3}=Ag_{23},\;\text{with}\;A=\frac{k_{\text{Ca}}}{k_{\text{Ca}}+3k_{-}+u}, \end{array}$$

and by summing Equations (11) and (12), we achieve a single ODE model for describing the dynamics of state variable *g*_2_ and *g*_3_ as follows:

(16) $$\begin{array}{c} \frac{dg_{23}}{dt}=-\left(2k_{-}\left(1-A\right)+uA\right)g_{23}+2k_{\text{Ca}}g_{1}. \end{array}$$

The corresponding Markov chain model takes values in the state space *S*={*G*_0_, *G*_1_, *G*_23_, *Y*} (Figure 1(c)) and is described by the following generating matrix, *M*_G_ap__:

(17) $$\begin{array}{c} M_{{\text{G}}_{\text{ap}}}=\left[\begin{array}{cccc} -3k_{\text{Ca}} & 3k_{\text{Ca}} & 0 & 0\\ k_{-} & -2k_{\text{Ca}}-k_{-} & 2k_{\text{Ca}} & 0\\ 0 & 2k_{-}\left(1-A\right) & -2k_{-}\left(1-A\right)-uA & uA\\ 0 & 0 & 0 & 0 \end{array}\right]. \end{array}$$

Note that state *Y* of the Markov chain described by *M*_G_ap__ is an absorbing state: the process can never leave *Y* after entering it, reflecting that fusion is an irreversible process. Then *M*_G_ap__ can be rewritten as

(18) $$\begin{array}{c} M_{{\text{G}}_{\text{ap}}}=\left[\begin{array}{cc} D_{3\times 3} & d_{3\times 1}\\ 0_{1\times 3} & 0 \end{array}\right], \end{array}$$

where

(19) $$\begin{array}{c} D_{3\times 3}=\left[\begin{array}{ccc} -3k_{\text{Ca}} & 3k_{\text{Ca}} & 0\\ k_{-} & -2k_{\text{Ca}}-k_{-} & 2k_{\text{Ca}}\\ 0 & 2k_{-}\left(1-A\right) & -2k_{-}\left(1-A\right)-uA \end{array}\right], \end{array}$$

describes only the transitions between the transient states *G*_0_, *G*_1_, and *G*_23_ and **d**=[0,0, *uA*]^*T*^ is a vector containing the transition intensities from the transient states to the absorbing state *Y*. The row vector **0** ∈ *ℝ* ^1×3^ consists entirely of 0's since no transitions from *Y* to the transient states can occur. The remaining element of the matrix *M*_G_ap__ is 0 and gives the transition rate out of the absorbing state.

Using phase-type distribution results for Markov chains [10], we obtain an explicit formula for calculating the expected event rate *λ*_*Y*_ to reach the absorbing state *Y*, given the initial probability row vector ***π*** for the transient states (***π***=(*π*_*G*_0__, *π*_*G*_1__, *π*_*G*_23__)), as

(20) $$\begin{array}{c} \lambda_{Y}=\frac{1}{\pi \left(-D^{-1}\right)1}, \end{array}$$

where **1** ∈ *ℝ* ^3×1^.

### 2.3. Granule-CaV Complex Model with 1 : 1 and 1 : *n* Stoichiometries

#### 2.3.1. 1 : 1 Stoichiometry

By coupling the CaV and exocytosis models, we obtain the 12-state Markov chain model of Figure 1(d). The model takes values in the state space

(21) $$\begin{array}{c} S=\left\{CG_{0},OG_{0},BG_{0},CG_{1},OG_{1},BG_{1},CG_{23},OG_{23},BG_{23},CY,OY,BY\right\}, \end{array}$$

and its transition matrix, *D*_G:CaV_, is as follows:

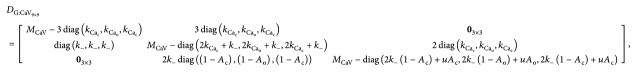
(22)
where *M*_CaV_ is defined by Equation (1), *k*_Ca_c__(*A*_c_) by Equation (8) (Equation (15)) with Ca_G_=Ca_c_, i.e., the concentration at the granule when the associated CaV is closed (or inactivated, i.e., Ca_c_=Ca_b_), and *k*_Ca_o__(*A*_o_) by Equation (8) (Equation (15)) with Ca_G_=Ca_o_, i.e., the concentration at the granule when the associated CaV is open, computed by Equation (4). Then, the expected exocytosis rate for the single granule, *λ*_*Y*_1__, can be estimated by using Equation (20), assuming initially the granule in state *G*_0_ and the CaV closed, i.e., the complex in the state *CG*_0_(***π***=(1, **0**_1×8_)), as

(23) $$\begin{array}{c} \lambda_{Y_{1}}=\frac{1}{\pi \left(-{D_{\text{G:CaV}}}^{-1}\right)1}, \end{array}$$

where **1** ∈ *ℝ* ^9×1^.

We also consider the particular case with non-inactivating CaV (i.e., the Ca^2+^ channel can be only in *C* or in *O*). In this case, *M*_CaV_ ∈ *ℝ* ^2×2^ and is defined by Equation (1) with *δ*=*γ*=0, and then *D*_G:CaV_, given by Equation (22), belongs to *ℝ* ^6×6^.

#### 2.3.2. 1 : *n* Stoichiometry

In the following, we assume the case where the granule is coupled with more than one CaV. In particular, by considering *k* Ca^2+^ channels, we have a Markov chain model with *n*_S_=∑_*i*=0_^*k*^(*k*+1 − *i*)=(*k* ^2^/2)+(3*k*/2)+1 possible states describing the *k* CaVs. In particular, the CaVs model takes values in the state space *S*={*C*_*k*−*i*−*j*_ *O*_*i*_ *B*_*j*_} with *j* ∈ {0,…, *k*} and *i* ∈ {0,…, *k* − *j*}, and its generating matrix, *M*_*k*CaV_, is given by

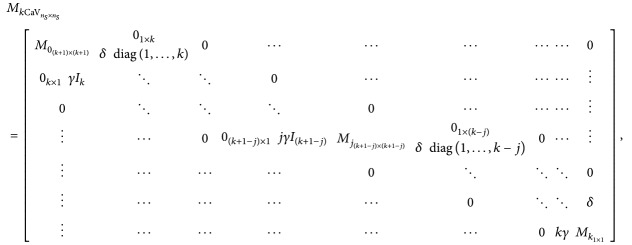
(24)where
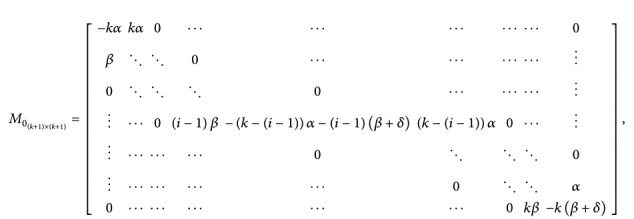
(25)

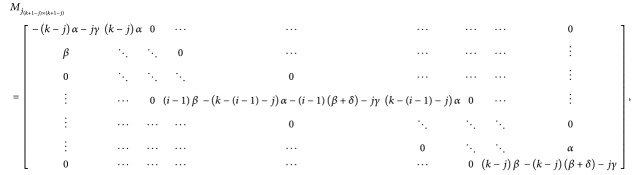
(26)and *M*_*k*_1×1__=−*kγ*.

Then, by coupling the CaVs and exocytosis models, we obtain a 4*n*_*S*_-state Markov chain model. The model takes values in the state space *S*={*C*_*k*−*i*−*j*_ *O*_*i*_ *B*_*j*_ *G*_*l*_,…, *C*_*k*−*i*−*j*_ *O*_*i*_ *B*_*j*_ *G*_23_, *C*_*k*−*i*−*j*_ *O*_*i*_ *B*_*j*_ *Y*}, with *j* ∈ {0,…, *k*}, *i* ∈ {0,…, *k* − *j*} and *l* ∈ {0,1}, and its transition matrix, *D*_G:kCaV_, can be written as
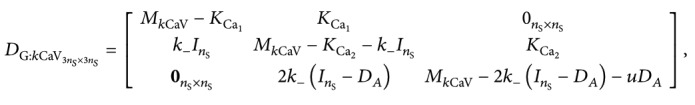
(27)where
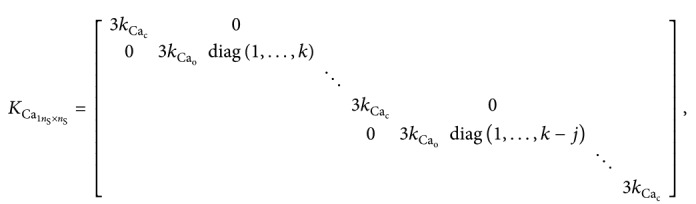
(28)

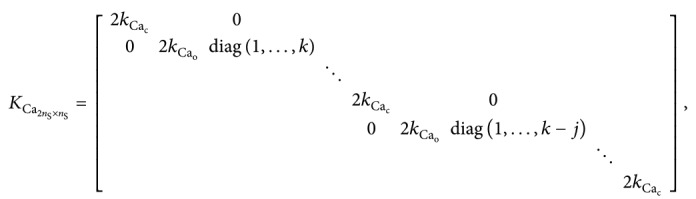
(29)

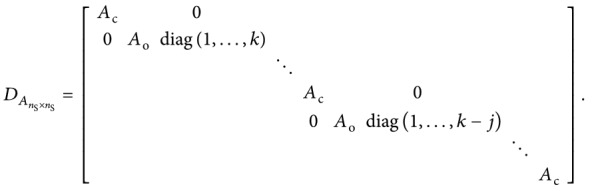
(30)


Then, the expected exocytosis rate for the single granule coupled with *k* CaVs, *λ*_*Y*_*k*__, can be estimated by using Equation (20), assuming initially the granule in state *G*_0_ and the *k* CaVs closed, i.e., the complex is initially in state *C*_*k*_ *G*_0_ ***π***=(1, 0_1×(3*n*_S_ − 1)_), which yields

(31) $$\begin{array}{c} \lambda_{Y_{k}}=\frac{1}{\pi \left(-D_{\text{G:}k\text{CaV}}^{-1}\right)1}, \end{array}$$

where **1** ∈ *ℝ* ^3*n*_S_×1^.

For the particular case with non-inactivating CaVs channels, *M*_*k*CaV_=*M*_0_ by Equations (24) and (25) with *δ*=*γ*=0, and then, *D*_G:*k*CaV_, given by Equation (27), belongs to *ℝ* ^3(*k*+1)×3(*k*+1)^.

In order to compare the rate for a granule coupled with different number *k* of CaVs, we define the relative rate, *ρ*_*λ*_*k*__, as

(32) $$\begin{array}{c} \rho_{\lambda_{k}}=\frac{\lambda_{Y_{k}}}{\lambda_{Y_{n}}}, \end{array}$$

with *k*=1,…, *n*. Moreover, in order to compare the rate at different distances from the granule to CaVs, we define the relative distance rate, *ρ*_*λ*_*d*__, as

(33) $$\begin{array}{c} \rho_{\lambda_{d}}=\frac{\lambda_{Y_{d}}}{\lambda_{Y_{d_{\min}}}}, \end{array}$$

where *λ*_*Y*_*d*__ is the rate computed at a given distance *r*_*G*_ and *λ*_*Y*_*d*_min___, the rate computed at *r*_G_=10 nm.

## 3. Results and Discussion

We analyze the behavior of the devised exocytosis model where the single granule is coupled with *k* Ca^2+^ channels by using phase-type distribution results for Markov chains [10] (see Methods). First, we assume that a granule is coupled with one CaV and, then, we extend the results to a more general case with *k* CaVs. Moreover, we consider for both the cases (1 or *k* CaVs) that the granule forms complexes with inactivating or non-inactivating CaVs. This scenario reflects, e.g., what it is observed in pancreatic beta-cells where the two main high voltage-activated Ca^2+^ channels, the L- and P/Q-type Ca^2+^ channels, are examples of inactivating and non-inactivating CaVs, respectively [20].

### 3.1. Granule Coupled with One Inactivating (or Non-Inactivating) CaV

**Figure 2:**
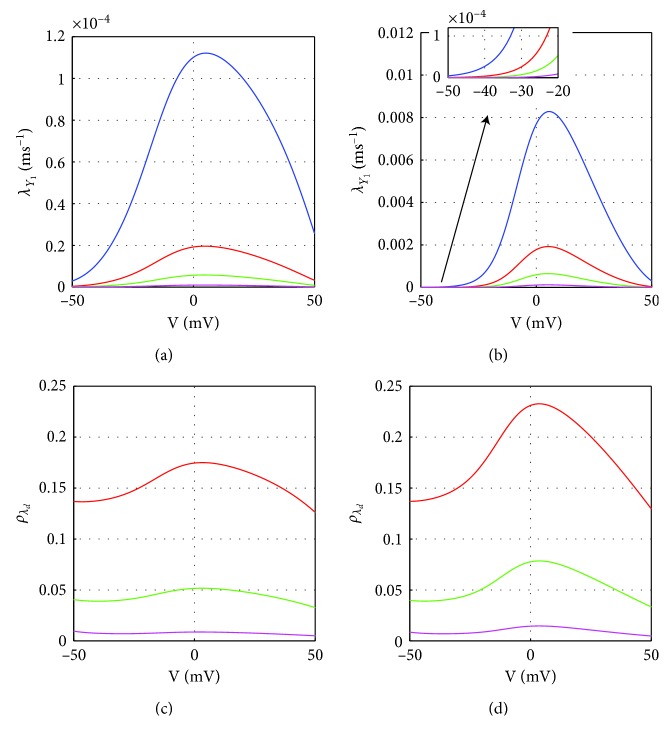
Expected exocytosis rate for single granule coupled with one (inactivating or non-inactivating) CaV. (a, b) Expected exocytosis rate *λ*_*Y*_1__ for the granule at different distances *r*_*G*_ from one inactivating (a) or non-inactivating (b) CaV: *r*_*G*_=10 nm (blue curves), *r*_G_=20 nm (red), *r*_G_=30 nm (green), and *r*_G_=50 nm (magenta). Note the different scales on the *y*-axes. The insert in (b) is a zoom on the lower, left part of the figure for comparison with (a). (c, d) Relative rate *ρ*_*λ*_*d*__ computed at different distances (*r*_G_=20 nm (red), *r*_G_=30 nm (green), and *r*_G_=50 nm (magenta)) of the granule from the inactivating (c) or not-inactivating (d) CaV and compared to the case with *r*_G_=10 nm.

Figure 2 shows the expected exocytosis rate, *λ*_*Y*_1__, computed by Equation (23), for a granule at different distances from an inactivating CaV channel. Independently of the distance to the CaV, the exocytosis rate has a bell-shaped relation to voltage, as seen experimentally [20–22]. The same holds true in the case of non-inactivating CaV (Figure 2). As the distance between the granule and the Ca^2+^ channel increases, the expected rate decreases substantially and nonlinearly (for instance, in Figure 2, compare the red and blue lines for *r*_G_=20 nm and *r*_G_=10 nm, respectively). This is clearer from Figure 2, showing the relative distance rate *ρ*_*λ*_*d*__ defined by Equation (33) for different values of *r*_G_. Note that increasing the distance by a factor of two corresponds to a more than five-fold reduction of the exocytosis rate (the relative ratio is less than 0.2, see the red plot in Figure 2). This steep dependence of the distance to the channel is because the calcium levels drop rapidly, moving away from the channel [8, 23].

We perform a similar analysis for the case where a granule is coupled with a non-inactivating CaV (Figure 2). We note an increase about of two orders of magnitudes for the exocytosis rate compared to the case with a granule coupled with an inactivating CaV (Figures 2 and 2): the exocytosis proceeds more rapidly since the triggering Ca^2+^ signal is increased due to non-inactivation of Ca^2+^ currents. Also in this case, the degree of decrease for the rate is much higher than the relative increase for the distance (Figure 2). However, the benefit in terms of *ρ*_*λ*_*d*__ by reducing the distance is slightly less than that obtained with inactivating CaV (compare Figures 2 and 2): for the case with inactivating CaV, it seems that moving away from the channel, *ρ*_*λ*_*d*__ decreases more due to the inactivation of CaV that determines a further drop of calcium levels.

### 3.2. Granule Coupled with *k* Inactivating (or Non-Inactivating) CaVs

**Figure 3:**
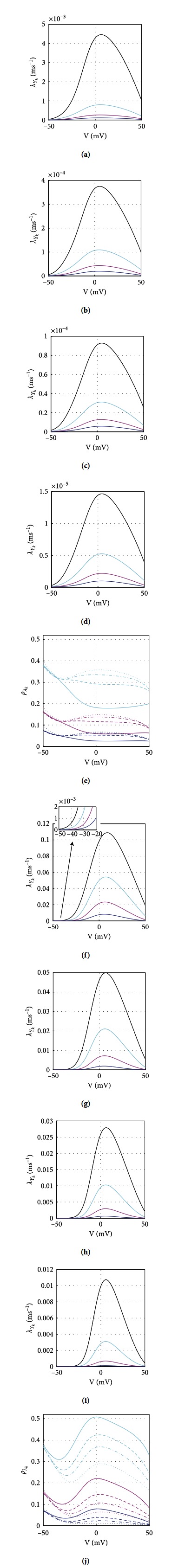
Expected exocytosis rate for single granule coupled with *k* (inactivating or non-inactivating) CaVs. (a–d) and (f–i) Expected exocytosis rate *λ*_*Y*_*k*__ for the granule at fixed distance *r*_G_ from *k* (*k*=1 (blue curves), *k*=2 (magenta), *k*=4 (cyan), and *k*=8 (black)) inactivating/not-inactivating CaVs: *r*_G_=10 nm (a/f), 20 nm (b/g), 30 nm (c/h), and 50 nm (d/i). The insert in (f) is a zoom on the lower, left part of the figure for comparison with (a). (e) and (j) Relative rate *ρ*_*λ*_*k*__ obtained from the granule coupled with *k* inactivating/not-inactivating CaVs (e/j), for *k*=1 (blue), *k*=2 (magenta), and *k*=4 (cyan) and compared to the case with *n*=8 CaVs. The different type of line corresponds to *ρ*_*λ*_*k*__ computed at fixed distance: *r*_G_=10 nm (solid line), *r*_G_=20 nm (dashed), *r*_G_=30 nm (dash-dotted), and *r*_G_=50 nm (dotted).

Figures 3–3 show the expected exocytosis rate *λ*_*Y*_*k*__ computed by Equation (31), for a granule coupled with different numbers of inactivating CaVs and at fixed distances between the granule and the CaVs. It is clear that increasing the number of CaVs coupled with the granule determines a rise of the exocytosis rate. Moreover, as the number of CaVs coupled with the granule increases, the rise in the rate is more pronounced when the distance of the granule from the CaVs is small. This is evident by considering the relative rate *ρ*_*λ*_*k*__ defined by Equation (32) (Figure 3). For instance, consider the cyan curves computed for *k*=4 with different types of lines denoting the different distances of the granule from the CaVs. In this case, the number of CaVs decreases by a factor of 2 (from 8 to 4) while the exocytosis rate drops more than threefold for *r*_G_=20 nm (dashed cyan line, *ρ*_*λ*_*k*__ < 0.3, for *V* > −10 mV) and more than fivefold for *r*_G_=10 nm (solid cyan line, *ρ*_*λ*_*k*__ < 0.2, for *V* > −10 mV).

As done for the case with one CaV, we performed the same analysis with *k* non-inactivating CaVs coupled with the granule (Figures 3–3). Also in this case, it is clear that increasing the number of CaVs determines a rise of the exocytosis rate for the granule. Surprisingly and in contrast with the case with inactivating CaVs, as the number of non-inactivating CaVs increases, the relative rise in exocytosis rate is much higher at larger distances from the CaVs, as shown in Figure 3 reporting the relative rate *ρ*_*λ*_*k*__. In case the number of CaVs is reduced from 8 to 4, the exocytosis rate decreases by 2–2.5-fold when the granule is near the CaVs (see the solid cyan curve for *r*_G_=10 nm, 0.4 < *ρ*_*λ*_*k*__ < 0.5 with −20  mV <*V* < 40 mV), while it goes down fivefold when the granule is far from CaVs (see the dotted cyan curve for *r*_G_=50 nm, 0.2 < *ρ*_*λ*_*k*__ < 0.3 with −20 mV <*V* < 40 mV). It seems that when the granule is surrounded by more non-inactivating CaVs, it is not necessary that the granule is very close to the CaVs for triggering exocytosis.

### 3.3. Relationship between Ca^2+^ Influx and Exocytosis

**Figure 4:**
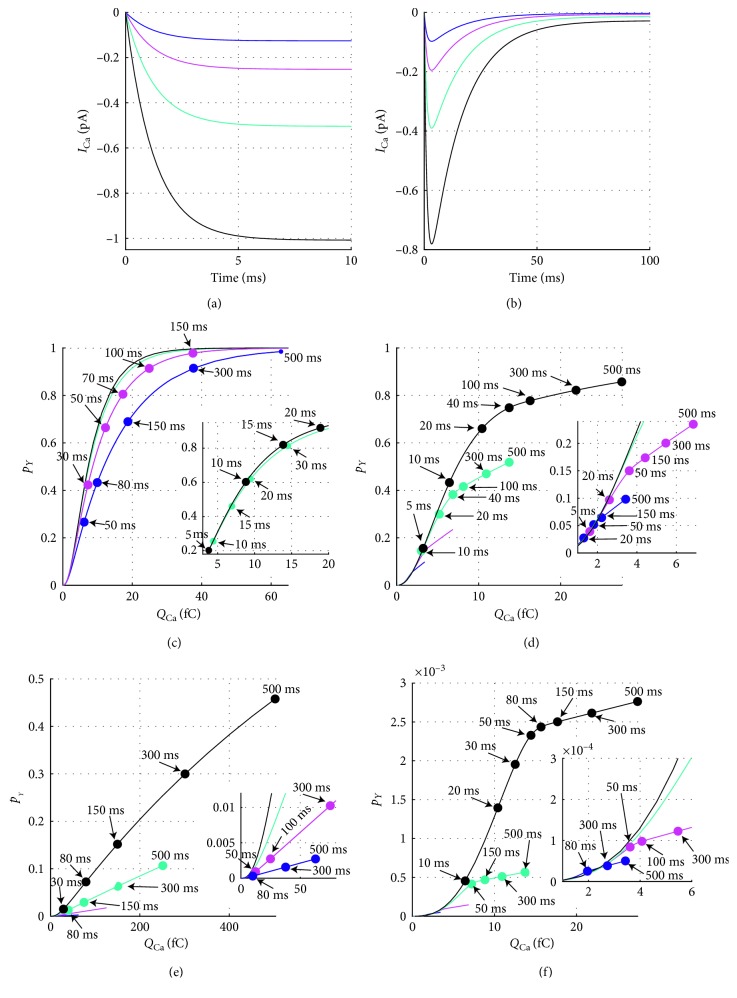
Relationship between Ca^2+^ influx and exocytosis rate. (a, b) Ca^2+^ current for the different number of non-inactivating (a) and inactivating (b) CaVs. (c, d) Probability of exocytosis, *p*_*Y*_, vs. integral of Ca^2+^ currents, *Q*_Ca_, computed at *V*=0 mV by increasing the integration time, *t*_s_, from 1 to 500 ms, for the granule at distance *r*_G_=10 nm from *k* not-inactivating (c) or inactivating (d) CaVs. (e, f) Legends as in (c, d) with *r*_G_=100 nm. In each panel, the different colors represent different number of CaVs: blue curve for *k*=1; magenta for *k*=2; cyan for *k*=4; and black for *k*=8. The inserts in (c–f) show a zoom-in of *p*_*Y*_ vs. *Q*_Ca_ on lower *Q*_Ca_ values for the granule coupled with different number *k* of CaVs: *k*=4 and 8 in (c) and *k*=1, 2, 4, and 8 in (d–f).

To investigate the relationship between exocytosis and Ca^2+^ loading, we consider a set of scenarios where the granule is coupled with different number of non-inactivating or inactivating CaVs, placed very close (10 nm) or far (100 nm) from the granule.  Figure 4 shows the calcium current at *V*=0 mV, for different numbers of non-inactivating CaVs, while Figure 4 shows the corresponding cases with inactivating CaVs. In the latter, it is evident how the calcium influx drops after few tens of ms due to the inactivation of the CaVs. Figures 4 and 4 show the probability of exocytosis *p*_*Y*_ (*p*_*Y*_=*P*(*S*(*t*)=*Y*)) vs. the integral of the Ca^2+^ current, *Q*_Ca_, defined by Equation (6), for the granule placed close to the CaV cluster, for different numbers of CaVs (*r*_G_=10 nm). For the case of non-inactivating CaVs (Figure 4), *p*_*Y*_ raises linearly with *Q*_Ca_, with slope that increases with the number of CaVs and then saturates due to the depletion of the granule pool as *p*_*Y*_ approaches 1 (see also [24]). For inactivating CaVs, we note a change of the slope of the linearity between *p*_*Y*_ and *Q*_Ca_ that is not only due to depletion (when *y* ≥ 0.5) but also to near-complete inactivation of CaVs, in particular after 50 ms (Figure 4). Figures 4 and 4 show *p*_*Y*_ vs. *Q*_Ca_ when the granule is placed far from CaVs (*r*_G_=100 nm). Due to the distance to CaVs, the calcium concentration at the granule increases only modestly; hence, a greater calcium influx *Q*_Ca_ is needed to allow the granule to move through the Markov chain from *N*_0_ to *Y* and undergoes exocytosis. This causes an evident initial delay for the granule to be released, resulting in an initial convex relation between *p*_*Y*_ and *Q*_Ca_. After this initial phase, for the case of non-inactivating CaVs (Figure 4), *p*_*Y*_ raises linearly with *Q*_Ca_ with slope depending on the number of CaVs. For higher *Q*_Ca_, the slope of *p*_*Y*_ slightly decreases in the case with *k*=8 CaVs reflecting slight depletion of the granule pool (*p*_*Y*_ ≈ 0.5 at *Q*_Ca_=500 fC). For inactivating CaVs (Figure 4), as for the case with *r*_G_=10 nm, we note a change of the linearity between *p*_*Y*_ and *Q*_Ca_ that is due to CaV inactivation.

## 4. Conclusions

In this paper, we devise a strategy that allows us to characterize the local interactions between granules and CaVs. The methodology is similar to our approach for modelling the local effect of CaVs on whole-cell BK currents [9]. We develop Markov chain models describing the dynamics of a single granule coupled with one or more inactivating (or non-inactivating) Ca^2+^ channels and use phase-type distribution results [10] for estimating the expected exocytosis rate.

We investigate how the release probability of a granule can be affected by varying the number of CaVs and the distance of the (Ca^2+^ sensor of the) granule from CaVs. In particular, from our analysis, we find that the distance between the granule and CaVs is a major factor in determining the exocytosis rate, as we recently demonstrated and quantified explicitly [23]. Further and in agreement with experiments [23], the simulations presented here show that the increase of the number of CaVs coupled with the granule determines a much higher rise of the exocytosis rate, which in the case of inactivating CaVs is more pronounced when the granule is close to CaVs (≈10 nm), whereas for non-inactivating CaVs the highest relative increase in rate is obtained when the CaVs are far from CaVs (≈50  nm).

We also study the relationship between Ca^2+^ influx and exocytosis. The results of the devised exocytosis model confirm that the granule secretion is generally linearly related to the integral of Ca^2+^current, as experimentally observed [25–29] and theoretically justified [24]. Surprisingly, for the case of inactivating CaVs, our analysis shows a change of the linear relation between *p*_*Y*_ and *Q*_Ca_ due to near-complete inactivation of CaVs. This fact is due to the rather complex exocytosis model where the efficacy of Ca^2+^ influx in triggering exocytosis depends on the number of active CaVs, as clearly seen in the case of non-inactivating CaVs (Figures 4 and 4), because of multiple steps of Ca^2+^ bindings before exocytosis. During inactivation, the effective number of CaVs declines, which has a similar effect as reducing the number of CaVs, and hence the slope of the relation between exocytosis and *Q*_Ca_ decreases. This finding reinforces the notion that a concave relation between exocytosis and Ca^2+^ influx does not necessarily reflect pool depletion [24] and provides a new example of such a scenario.

## Figures and Tables

**Table 1:** Model parameters.

Parameter	Value	Unit
CaV model parameters		
*α*_0_	0.6	ms^−1^
*α*_1_	−0.1	mV^−1^
*β*_0_	0.2	ms^−1^
*β*_1_	0.0375	mV^−1^
*γ*	0.002	ms^−1^
*δ*_0_	0.0025	*μ*M^−1^·ms^−1^

Parameters for calculating Ca^2+^ concentration at different distances		
*r*_Ca_	7	nm
*r*_G_	10, 20, 30, 50	nm
*D*_Ca_	250	*μ*m^2^·s^−1^
*F*	9.6485	C mol^−1^
*k*_B_	500	*μ*M^−1^·s^−1^
*B*_total_	30	*μ*M
*V*_Ca_	60	mV
${\bar{g}}_{\text{Ca}}$	2.8	pS
Ca_c_	0.1	*μ*M
Ca_b_	0.1	*μ*M

Exocytosis model parameters		
*k*_+_	1.85	*μ*M^−1^·s^−1^
*k*_−_	50	s^−1^
*u*	1000	s^−1^

## Data Availability

No data were used to support this study.
